# Change in the Healthiness of Foods Sold in an Australian Supermarket Chain Following Implementation of a Shelf Tag Intervention Based on the Health Star Rating System

**DOI:** 10.3390/nu14122394

**Published:** 2022-06-09

**Authors:** Adrian J. Cameron, Amy Brown, Liliana Orellana, Josephine Marshall, Emma Charlton, Winsfred W. Ngan, Jaithri Ananthapavan, Jasmine Isaacs, Miranda Blake, Gary Sacks

**Affiliations:** 1 Global Obesity Centre, School of Health and Social Development, Deakin University, Geelong, VIC 3220, Australia; josephine.marshall@deakin.edu.au (J.M.); emma.charlton@telethonkids.org.au (E.C.); wwtngan@gmail.com (W.W.N.); jaithri.ananthapavan@deakin.edu.au (J.A.); jasminejisaacs@gmail.com (J.I.); miranda.blake@deakin.edu.au (M.B.); gary.sacks@deakin.edu.au (G.S.); 2 City of Greater Bendigo Council, Bendigo, VIC 3550, Australia; amybrownprofessional@gmail.com; 3 Biostatistics Unit, Faculty of Health, Deakin University, Geelong, VIC 3550, Australia; l.orellana@deakin.edu.au

**Keywords:** supermarket, intervention, shelf tag, nutrient profiling

## Abstract

Introduction: Most people in Australia buy most of their food in supermarkets. Marketing techniques promoting healthy foods in supermarkets can be important to encourage healthy eating at a population level. Shelf tags that highlight the healthiness of products have been identified as one such promising initiative. The aim of this study was to assess changes in the healthiness of foods sold in an Australian supermarket chain following implementation of a shelf tag intervention based on the Australian Health Star Rating (HSR) system. Methods: A controlled, non-randomised trial was undertaken in seven supermarkets (intervention: *n* = 3; control: *n* = 4) of a single chain in Victoria, Australia, over 12 weeks (4 weeks baseline, 8 weeks intervention period) between August and November 2015. The intervention involved provision of a shelf tag indicating the HSR of all packaged products that scored 4.5 or 5 stars (‘high-HSR products’) using the Australian HSR system. Posters indicating the healthiness of fresh fruits and vegetables (not eligible for an HSR rating, as they are not packaged) were also installed. Weekly per store sales data were provided by the retailer. In an intention-to-treat analysis (with intervention status of individual products based on their eligibility to be tagged), the proportion (%) of all ‘high-HSR’ packaged food sold and the volume of key nutrients (saturated fat, total fat, sodium, total sugar, protein, carbohydrates and energy) per 100 g sold were assessed. Difference-in-difference analyses were conducted to determine the difference between intervention and control stores in terms of mean outcomes between baseline and intervention periods. Customer exit surveys (*n* = 304) were conducted to evaluate awareness and use of the shelf tags and posters. Results: The proportion of ‘high-HSR products’ sold increased in the intervention period compared to the baseline period in each of the three intervention stores (average increase of 0.49%, 95% CI: −0.02, 0.99), compared to a decrease of −0.15% (−0.46, 0.15) in control stores (*p* = 0.034). The overall increase in intervention compared to control stores (difference-in-difference) of 0.64% represents an 8.2% increase in the sales of ‘high-HSR products’. Sales of total sugar, total fat, saturated fat, carbohydrates, sodium, protein and total energy in packaged food all decreased significantly more in intervention stores compared to control stores. Sales of fresh fruits and vegetables decreased in intervention stores compared to control stores. Customer surveys found that 34.4% noticed the shelf tags. Of those who noticed the tags, 58% believed the shelf tags influenced their purchases. Conclusions: With this study, we found that the use of shelf tags that highlight the healthiest packaged foods in a supermarket setting showed promise as a mechanism to improve the healthiness of purchases. Opportunities to scale up the intervention warrant exploration, with further research needed to assess the potential impact of the intervention on overall population diets over the longer term.

## 1. Introduction

With supermarkets accounting for around half of all food and grocery spending globally, they are a key component of the food environment and represent an opportunity to promote healthier food purchases at a population scale. Marketing techniques modifying elements of product, price, promotion and placement in an effort to encourage consumers to buy healthier food can have an important impact on the healthiness of food purchased [1,2,3]. Unhealthy foods, which should only constitute a small fraction of the overall diet, are frequently the focus of marketing efforts [4,5,6,7], although the potential to promote a healthy diet using the same methods has been increasingly recognised [1,8,9].

Initiatives designed to encourage healthy eating in the supermarket setting have utilised a diverse range of marketing strategies, including shelf tags, product placement, mass media, signage, price discounts and taste testing. A recent review of 50 studies in the supermarket setting using non-price-based marketing techniques revealed that shelf tags highlighting the healthiness of products was a particularly promising intervention strategy [10].

In that review, 14 of the 17 studies that assessed some form of shelf labelling reported favourable outcomes (e.g., increased sales of targeted products). Ten studies examined the impact of shelf labelling alone or as the primary focus of the intervention. Five of these ten studies were published more than twenty years ago and used shelf labels highlighting foods with specific nutritional properties (e.g., low in fat, sodium or cholesterol) [11,12,13,14,15]. Five studies in the review [10] were published after 2010 (plus five published since the review) and examined the impact of the Guiding Stars^®^ (Scarborough, ME, USA) and (now discontinued) NuVal^®^ nutrient profiling systems [16,17,18,19,20,21,22,23,24] or used these systems to guide shelf tag placement [25]. Both of these North American systems incorporated a small logo into the price tag (0–3 stars for Guiding Stars^®^ and a score of 0–100 for NuVal^®^), with all five studies reporting significant increases in purchases of healthier foods. Most recently, a natural experiment in Belgium testing the effect of adding the voluntary European NutriScore to electronic price labels was evaluated, with small effects on sales of healthier products observed, albeit only for certain food categories [26]. No studies have been conducted using other nutrient profiling systems, such as the Australian/New Zealand Health Star Rating system.

Shelf tags incorporating summaries of nutrient profiling have the potential to impact sales of both healthy and unhealthy foods [10]. Few studies testing the effect of this type of shelf tag have been undertaken in real-world supermarkets, with none in Australia and none using more prominent “specials-style” shelf tags [10]. Experimental studies testing the impact of shelf tags on store sales and the nutrient composition of food purchased are important to inform retailer policy and practice and encourage healthier food environments.

The aim of the present study was to assess the changes in the healthiness of foods sold in an Australian supermarket chain (three intervention and four control supermarkets) following implementation of an eight-week shelf tag intervention in Victoria, Australia. The intervention promoted healthier products using the Australian and New Zealand Health Star Rating (HSR) system, which was introduced in Australia in 2014 [27].

## 2. Methods

### 2.1. Study and Timeline

The study was one of three short-term controlled trials conducted to test the effect of healthy eating interventions in the supermarket setting [28] and was conducted in collaboration with a supermarket retailer (Champions IGA, Bendigo, Australia), the City of Greater Bendigo and the Victorian Health Promotion Foundation (VicHealth, Melbourne, Australia). Tested interventions tested were codesigned through a series of meetings between academic, local government and retail partners and were based on the following criteria: low cost, feasible (for the retailer) and scalable, not likely to be detrimental to retailer profits and likely to improve diets at a population level. The study was a non-randomised, controlled trial of HSR shelf labels in seven supermarkets, with a four-week baseline period in August–September 2015 and an eight-week intervention period between September and November 2015. The number of supermarkets included in the study and the length of the baseline and intervention periods were determined in conjunction with the supermarket retailer, taking into account their operational constraints and data availability. The intervention period was designed to be longer than the baseline period in order to maximise the impact of the intervention on behaviour change within the available time for conducting the study.

### 2.2. Health Star Rating System

The HSR system is a front-of-pack labelling system funded and endorsed by Australian federal, state and territorial governments and developed collaboratively with the food industry, public health, Food Standards Australia New Zealand and consumer groups. It was designed to simplify the nutritional information provided on food packaging into a single score, with the interpretive label making it easier to quickly and effectively inform healthy food choices [27]. Using the system, packaged food can be rated between 0.5 (least healthy) and 5 stars (most healthy) in 0.5-point increments. At the time of the trial, the HSR was not applicable to food that did not come in standardized packaging (e.g., fresh fruit, vegetables, meat, fish, deli items, etc.). The system is voluntary; in 2019, it was reported that 41% of eligible products displayed the logo [29], with Australia’s largest supermarket retailers committing to including the HSR on all packaged foods [30]. A 2018 report on consumer perceptions of HSR noted that 76% of respondents found the system easy to both use and understand [31]. The HSR algorithm takes into account both positive nutrients/attributes (e.g., fruit, vegetable, nut and legume content; fibre and protein) and risk-associated nutrients linked to obesity, cardiovascular diseases and type 2 diabetes (e.g., saturated fat, total sugar and sodium). The HSR is calculated differently depending on the food group (i.e., non-dairy beverages, dairy beverages, cheese, all other dairy, oils and spreads and all other food), with the score being designed for comparisons between products in the same product category. HSR for this study was calculated using the original algorithm, which has since been modified following a review of the system. Full details with respect to calculation of the Health Star Rating can be found on the HSR website: http://www.healthstarrating.gov.au (accessed on 10 April 2022).

### 2.3. Stores and Setting

The participating supermarkets (3 intervention and 4 control stores) are part of the Champions IGA group, which had common pricing, marketing and promotion strategies across these seven stores, all located in regional Victoria, Australia, during the study period. The three intervention stores were selected by the retailer based on proximity to the retailer headquarters (in Bendigo), with two located in Bendigo (pop. ~100,000) and one in the nearby town of Heathcote (pop. <3000). The four control stores were located in Geelong (2 stores, pop. ~175,000), Darley (pop. ~8000) and Whittlesea (pop. ~5000). As part of the same chain, sales patterns of control and intervention stores varied over time in a tightly correlated manner (Supplementary File S2), although the intervention stores were located in more disadvantaged neighbourhoods as measured by the Australian Bureau of Statistics’ Index of Relative Socioeconomic Disadvantage (IRSD) (average of 14th percentile in Australia for intervention stores and 56th percentile for control stores. A low IRSD score indicates relatively greater disadvantage). At the time of the trial, IGA was the fourth largest supermarket chain in Australia (~1400 stores and 10% market share) and had more supermarkets in the Bendigo region (*n* = 9, all large stores) than any of the other three major Australian retailers (Coles, *n* = 3; Woolworths, *n* = 4; Aldi, *n* = 4).

### 2.4. Intervention

**Figure 1:**
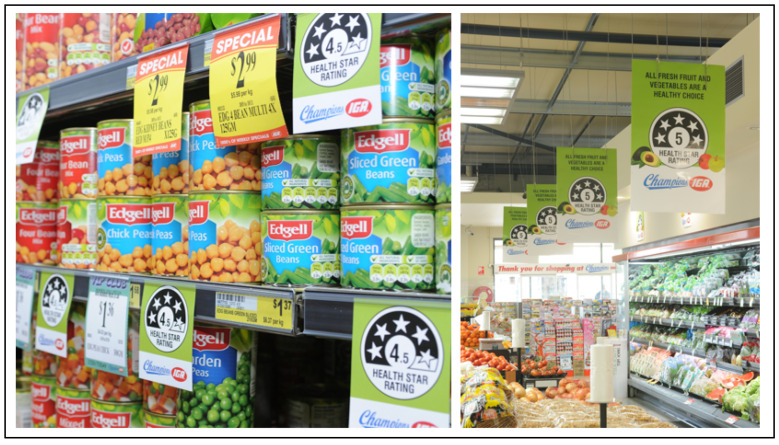
Example of Health Star Rating shelf tags and posters used to promote healthy eating in supermarkets.

The HSR of all packaged food (*n* ~ 20,000 products) sold by Champions IGA at the time of the study was identified using the 2015 Australian FoodSwitch database [32,33]. FoodSwitch is a database of packaged food and beverage products, including nutrition information, available from four large supermarkets [34]. The data are obtained using standardized collection methods and supplemented with crowd-sourced information for additional products [32,33]. The database included the HSR when it was included on product packaging, and when this was not available, it was calculated from nutrition information on the package, with category averages imputed for fibre, as well as fruit, vegetable, nut and legume content where not available. All products that were identified as having a 4.5- or 5-star rating (approximately 700–800 products per store or 6–7% of all packaged foods, including both products with a HSR logo on the pack and those for which the HSR was calculated for the study) were labelled with a shelf tag indicating their HSR (Figure 1). A cut-off point of 4.5 stars was chosen based on the number of products classified (i.e., the number classified could feasibly accommodate shelf tags in the store) and the desire to highlight the healthiest packaged foods. Shelf tags (equivalent in size to common “specials” tags—7 × 10 cm) were placed on the shelf directly in front of the products by research staff (Figure 1). For each intervention supermarket, we monitored products that had a shelf tag installed, products where shelf space was insufficient to fit a shelf tag and products that could not be found in the store (although they were in the sales database from that store). Shelf tags were monitored weekly by research staff and replaced where necessary (average of 32 tags replaced per store per week). Each intervention and control store had a varying range of products; for this reason and because shelf-tag application was not perfectly implemented for the reasons described above, a slightly different range of products received the intervention in each intervention store. Because fresh fruit and vegetables are not packaged and are therefore ordinarily excluded from the HSR scheme, we placed a series of large posters in the produce department that included the slogan, “All fresh fruit and vegetables are a healthy choice”, along with a prominent 5-star HSR logo (Figure 1). During data cleaning, it was discovered that due to an administrative error, some products were misclassified according to the HSR and mislabelled (total percentage of incorrectly classified products = 3.4%).

### 2.5. Outcome Measures

Weekly electronic sales data for each store were provided by the retailer. The time series data included the number of units of each product sold in a given week. Weight/volume of packaged products was calculated from product descriptions and for fresh produce from the AUSNUT 2011–13 Food Measures file [35]. The primary outcome of the study was units (and weight) of 4.5–5-star packaged products sold (“high-HSR products”) as a proportion of all packaged food sold in a week.

Secondary outcomes included units of high-HSR products sold in broad categories of packaged food as a proportion of all packaged food sold in that category (categories included “bread and bakery products”, “cereal and grain products”, “dairy”, “fish and fish products”, “packaged fruit and vegetables” and “non-alcoholic beverages”); volume (weight in kg) of fresh fruits and vegetables sold as a proportion of volume of all food sales; and the weight of nutrients sold required by law to be displayed on product packaging (protein, total fat, saturated fat, carbohydrate, total sugar and sodium) as a proportion of the total volume of food sold; and energy as kilojoules (kJ) per 100 g food sold. The nutrient content of fibre, calcium and cholesterol was not considered because it was voluntarily displayed on packaging and available for less than 30% of products analysed. Nutrient composition of all foods was obtained from the 2015 Australian FoodSwitch database and the AUSNUT 2011–13 food nutrient database [35]. Nutrient composition was obtained for 99.4% of products, with the rest excluded from nutrient-related analyses. Product weight accounted for the form specified on the nutrition information panel. For example, for cordial, nutrient information was based on the product as prepared with water; for ice cream, package size was reported in litres, and volume was calculated in grams; for fresh fruit and vegetables, weight was calculated as the edible portion.

### 2.6. Customer Perceptions

To evaluate the extent to which shelf labels on packaged foods and posters in the produce department were noticed by customers and whether customers felt it impacted their purchasing habits, we conducted exit surveys with a convenience sample of customers in each of the intervention stores during the last three weeks of the intervention at different times and on different days of the week. We aimed to recruit 100 customers per intervention store. Customers were asked whether they noticed the shelf labels, whether they noticed the posters and whether they felt that these had influenced their purchasing behaviour over the previous month (yes/no responses) (see Supplementary File S1 for customer survey). Basic demographic information and self-reported height and weight were also collected.

### 2.7. Statistical Analysis

To test the impact of the intervention on sales of ‘high-HSR products’ (the primary outcome), we conducted a difference-in-difference analysis. For each store, we calculated the average of the weekly percentage of ‘high-HSR products’ (relative to total weekly unit sales of packaged foods) sold during the baseline (four weeks) and intervention (eight weeks) periods, as well as the difference between intervention and baseline periods. The Kruskal–Wallis test and *t*-test were used to compare the difference-in-difference between the 3 intervention and 4 control stores. This approach was used to summarize the time series data available for each store in order to avoid (1) the week-to-week variability in sales of ‘high-HSR products’ within stores (due to other changes in the supermarket environment) and between stores (due to customer profile and store size); and (2) the short period covered by the time series (12 weeks), which made estimations based on segmented regression methods highly unreliable. The same approach was used to assess the effect of the intervention on the nutrient content of packaged foods (energy, protein, total fat, saturated fat, carbohydrate, total sugar, total sodium and energy density) sold and the impact of posters in the fresh produce department on the volume of fresh fruits and vegetables sold as a percentage of all food sales.

An intention-to-treat approach was adopted, whereby all products in intervention stores with an HSR rating ≥4.5 were considered to be tagged as ‘high-HSR products’, despite the fact that there was not space to install a tag for some products and the actual HSR classification used in the stores was not always 100% accurate (see Section 2.4). A per-protocol approach (analysis based on only products that were tagged as ‘high-HSR products’) was not feasible because of the challenges associated with deciding which products to include in intervention stores and control stores, which all have a distinct range of products. Descriptive statistics are reported for customer perception outcomes.

## 3. Results

Almost 2.2 million packaged food items with HSR data were sold across the seven supermarkets during the 4-week baseline and 8-week intervention periods. Of these, 176,543 (8.04%) were rated as either 4.5 or 5 stars. Of all unit sales in the three intervention stores where the product was eligible to receive a 4.5 or 5-star shelf tag, 76.4% were for products where a shelf tag had been installed, 12.8% were for products where a tag was not installed (due to insufficient space) and 10.8% were for eligible products that could not be located in the store. The product categories with the most 4.5- or 5-star-rated products sold during the study period were dairy (21.9%); frozen vegetables (14.7%); fruit and vegetable juices (14.6%); breakfast cereals (10.6%); and pasta, noodles and rice (5.8%).

### 3.1. Impact on Sales: Primary Outcome

The percentage of units of ‘high-HSR products’ sold (as a percentage of all packaged food sold) increased between the baseline and intervention period in each of the three intervention stores (by 0.32%, 0.42% and 0.72%, respectively), whereas in each of the control stores, it decreased (−0.42%, −0.17%, −0.03% and −0.01%, respectively) (Table 1). The average difference-in-difference between intervention and control stores was 0.64% (95% CI: 0.26%, 1.03%; *p* = 0.034 for Kruskal–Wallis test; *p* = 0.008 for *t*-test).

### 3.2. Impact on Sales: Secondary Outcomes

The change in sales of ‘high-HSR products’ for several categories of packaged foods was also investigated (as a percentage of all packaged food sold in each category), with no difference observed for “bread and bakery products”, “cereal and grain products”, “dairy”, “fish and fish products”, “packaged fruit and vegetables” and “non-alcoholic beverages” in intervention and control stores between the baseline and intervention periods (*t*-tests and K–W tests, both *p* > 0.05). The volume of fresh fruit and vegetables sold (relative to all food sold) decreased in intervention stores relative to control stores during the intervention, with an increase in control stores (0.63%, 95% CI: −0.1, 1.36) and a decrease in intervention stores (−1.16% (−2.18, −0.14), *p* = 0.034).

The volume of nutrients sold (grams per 100 g of packaged food sold) and total energy (kJ per 100 g of packaged food sold) decreased significantly in intervention stores compared to control stores for protein, total fat, saturated fat, sodium, carbohydrates, total sugar and total energy (*p* = 0.034 for all) (Table 2). For all nutrients, the changes in each of the intervention stores were greater than the change in any of the control stores.

### 3.3. Customer Perception Results

Of 304 intervention store customers who completed questionnaires, 78% were female, 25% were aged <40 years, 40% were aged 40–64 years and 35% were aged 65 years or older, with 22% having completed a university degree, 45% having completed high school and 33% having not completed high school. The mean self-reported body mass index (BMI) was 25.6 kg/m^2^, with 58.9% having a BMI in the overweight or obese range (>25 kg/m^2^). Eighty percent were regular shoppers at the store at which they were surveyed, and 57% reported being familiar with the HSR system. The percentage of those who noticed the HSR shelf tags in the store was 34.4%, with no differences in terms of age or gender but a slightly larger proportion among those classified as overweight or obese compared to those with normal weight (36% vs. 27%). A proportion of 35% of participants reported seeing HSR posters in the fresh food section, with 24% of respondents noticing both the posters and shelf tags. Of those who noticed shelf tags or posters, 58% believed that they had influenced their purchasing behaviour, with no difference in terms of age, sex or body weight category.

## 4. Discussion

This controlled trial provides evidence that a simple nutrient profiling intervention applied to shelf tags can improve the health profile of packaged food purchased in a real-world supermarket setting. Compared to control stores, the proportion of high-HSR products (those with an HSR rating ≥4.5) sold increased during the intervention period in each of the three intervention stores, with the increase being largely consistent over the study period. The amount of energy, saturated fat, total sugar and sodium per 100 g of packaged foods sold also improved in intervention stores compared to control stores. The intervention was shown to be popular among customers.

Although the intended use of the HSR system is on the front of packages, in this trial, we tested the efficacy of selectively applying ratings to the healthiest products and using much more visible shelf tags that were of an equivalent size and prominence to “specials” labels typically applied in Australian supermarkets. The application of the HSR to product packaging is voluntary (by manufacturers), whereas shelf tags can be used by retailers to apply a rating to all products. In this trial, we applied tags to all eligible products for which we had data (even if an HSR label was not displayed on the product) and where space for a shelf tag existed. The application of shelf tags to products with an HSR of 4.5 or greater was designed to provide a positive selection bias by promoting only the healthiest packaged foods. Non-HSR shelf tags are typically applied to specific products to highlight price promotions.

The HSR system is useful for comparing the nutrient profile of products within a single food category; however, because the algorithm used to calculate the rating depends on the food category (i.e., dairy beverages, cheese and processed cheese, other dairy products, non-dairy beverages, oils and spreads and everything else), it is difficult for shoppers to compare ratings for products in different groups (although in practice, shoppers may do this anyway due to a lack of detailed knowledge of the system) [36]. In addition, without data on the presence or absence of the HSR logo on each product, we were not able to test the interaction between our intervention and on-pack labels.

Previous studies that examined the effect of applying nutrient profiling to shelf tags [16,17,18,19,20,21,22,23,24,25,26,37] mostly reported outcomes that favoured healthier purchasing. Of these studies, one was an online experiment [19]; two were uncontrolled, interrupted time-series trials [17,18]; one compared purchases between frequent shopper card holders and Nielsen panel members [16]; and four only examined sales of single product categories (e.g., yoghurt and cereals) [20,21,23,37]. A study by Hobin et al. reported the effect of the Guiding Stars^®^ shelf labelling system in a large Canadian supermarket chain [22], whereas a study by Vandevijvere et al. examined the application of the NutriScore nutrient profiling logo to electronic labels in a Belgian supermarket chain [26]. Both were natural experiments wherein the retailer implemented the system in some stores but not others and involved systems where all products (regardless of rating) had logos applied. Like the current study, both also used a difference-in-difference analysis with an intention-to-treat approach. In the Canadian study, a small but significant change in purchasing of products with higher (healthier) star ratings was observed (an increase of 1.4% in mean star rating of products). Sales of one and three-star-rated products increased, and sales of zero- and two-star-rated products decreased. A positive impact on purchasing of total sugar, trans fat, fibre and omega-3 fatty acids was observed, but no change was evident for total calories, sodium, saturated fat or protein. Few customers understood (8.7%), reported using (2.0%) or trusted the Guiding Stars system (mean score of 2.8 on a 5-point scale). Findings from the Belgian study were also equivocal, with statistically significant increases in sales of both B-rated (healthier) and D-rated (less healthy) products (0.11% ±0.04 (S.E) and 0.12% ±0.04, respectively), decreases in C-rated (moderately healthy) products (−0.06 ±0.03) and no significant change in A-rated (healthiest) or E-rated (least healthy) products [26]. An important difference between these previous studies and our own study is that the nutrient profiling logos applied to price labels in both previous studies were relatively small and applied to all products (i.e., the intervention was educational rather than promotional). In comparison with the Canadian study of Hobin et al., a considerably higher proportion of customers both noticed and made use of the shelf tags in the current study. This may be explained by the high visibility of the shelf tags in this study in comparison with the much smaller Guiding Stars logo added to price tickets, as well as the higher profile of the national and government-endorsed HSR scheme in comparison to the Guiding Stars system, which was only used in a small number of supermarket chains.

At the time of this study, fresh fruit and vegetables were excluded from the HSR scheme, meaning that we were not able apply shelf tags for this large category of healthy food. We did, however, position posters throughout the fresh produce section to promote these products as a healthy choice. These posters were expected to have only a minimal (and positive) impact on purchasing of fruit and vegetables. The unexpected finding of a decrease in fruit and vegetable sales in intervention stores compared to control stores may be due to other (unmeasured) factors differentially impacting intervention and control stores at the time of the study, but it is also possible that it could reflect a substitution effect, whereby customers were purchasing more healthy packaged food but less fresh fruit and vegetables. Longer-term studies testing the effects of shelf tags on store purchasing of different food categories are required to clarify this issue, as well as effects within individual product categories.

## 5. Sustainability and Scalability of the Intervention

Our systematic review of the literature [10] found that most previous supermarket healthy eating initiatives have included price discounts or educational interventions, with their relatively high cost (e.g., for subsidising healthy food, or labour-intensive education campaigns) and the potential for unintended compensatory behaviour (with price discounts) presenting concerns around long-term feasibility, sustainability and scalability. In contrast, the intervention implemented in the present study is potentially low-cost and scalable and can be applied supermarket-wide. Supermarket interventions applied at scale have been found to be hugely cost effective, given their population-wide impact [38]. Shelf tags and posters have trivial production costs and take little time to install, with ongoing maintenance of shelf tags by staff likely the largest cost and greatest inconvenience for the retailer. The incorporation of nutrient profiling scores into the retailer sales management database (as seen in the Belgian NutriScore intervention [26]) is likely to lead to significant efficiencies in the implementation of a shelf tag intervention. The same strategy would allow nutrient profiling logos to be included on price tickets, which could be an intervention in its own right (albeit far less prominent than shelf tags), or to aide in the identification of products requiring a promotional shelf tag.

## 6. Strengths and Limitations

Key strengths of this study include the study design, with all intervention and control stores from the same chain, as well as the analysis of both store sales data and change in the volume of nutrients sold. Limitations include the small number of stores included, the short (eight week) intervention period and the fact that not 100% of eligible products could be tagged (due to space constraints and not being able to find the product). Although some front-of-pack labelling studies suggest that customers may find it easier to compare the nutrient profile of products if all products have a nutrient profile present [39], this was not feasible for the shelf tags used in the current study, given the space they take up. The lack of randomisation is also a potential limitation, with randomisation to intervention and control conditions not possible due to retailer preference. Despite this, purchasing patterns in control and intervention stores were shown to be highly comparable because the non-seasonal factors that impact purchasing (e.g., price, promotion and advertising) were held constant across stores in this chain (see Supplementary File S2). The fact that intervention stores were in considerably more disadvantaged neighbourhoods is a promising sign that this initiative could be effective in populations that are typically more difficult to reach [40,41]. The fact that those with lower incomes are constrained in their choices by the price of food could mean that this study may have underestimated the true effect of the intervention on the general population. Future studies are clearly needed to test the effect of the intervention in other contexts. The use of store sales data to measure primary outcomes is a strength in that such data are objective, accurate and comprehensive representing the entire population of shoppers. In the present study, we did not assess the impact on individual purchasing patterns, although future studies that use loyalty card data could achieve this. The impact of shelf tag interventions on population diets is also unknown and should be the subject of future investigations.

As a short-term trial, it is possible that this intervention applied over a longer period would see customers either increasingly use the shelf tags and base their purchasing decisions on them or reveal that their effect over time would diminish due to overfamiliarity. Longer-term trials are required to test this empirically. Some evidence exist that suggests that customers are willing to pay 3.7% more than the base price of a product for a product with an HSR tag [42].

## 7. Conclusions

Whereas additional studies are required to confirm the effects of HSR shelf tags over an extended period, the results of this 8-week intervention indicate that this promising supermarket intervention has the potential to impact food choice at a population level. The impact on nutrients purchased and customer perceptions suggest that this intervention is worthy of further and longer-term investigation. Being cheap and simple to install and requiring little effort by retailers, promotional shelf tags that utilise nutrient profiling systems could be eminently scalable, and this warrants further examination. This study has made important contributions to the literature by examining the efficacy of nutrition-related shelf tags applied only to the healthiest products storewide from a public health perspective and testing the application of the HSR system as a promotional tool on shelf tags rather than for front-of-pack labelling (as it was designed). Due to the position of supermarkets in the food system, relatively small changes in such settings can lead to changes in purchasing behaviours at the population level. The results of this trial support the concept of nutrient profiling systems, such as the HSR, which have the potential to underpin a coherent set of retailer policies for improving population diets that could include both food labelling and in-store marketing interventions.

## Figures and Tables

**Table 1:** Mean percentage of units of ‘high-HSR products’ sold (relative to the number of units of all packaged food sold) in control and intervention supermarkets.

Supermarket	% “High-HSR Products” Sold		
	Baseline Period	Intervention Period	Difference
Control store 1	8.48	8.06	−0.42
Control store 2	6.60	6.43	−0.17
Control store 3	7.00	6.97	−0.03
Control store 4	9.32	9.31	−0.01
Intervention store 1	7.46	7.78	0.32
Intervention store 2	8.96	9.38	0.42
Intervention store 3	6.89	7.60	0.72

**Table 2:** Mean difference between post- and pre-intervention in volume of nutrients sold from packaged foods as a percentage of volume of food sold in control and intervention supermarkets, as well as mean change in energy (kJ/100 g) sold.

Nutrient	Intervention Stores Mean Difference (95% CI)	Control Stores Mean Difference (95% CI)	*p*-Value ^1^
Protein	−0.14 (−0.4, 0.11)	0.16 (−0.29, 0.6)	0.034
Total fat	−0.64 (−0.91, −0.38)	0.3 (−0.55, 1.14)	0.034
Saturated fat	−0.25 (−0.33, −0.17)	0.22 (−0.11, 0.55)	0.034
Sodium	−0.01 (−0.03, 0)	0 (−0.02, 0.03)	0.034
Carbohydrates	−0.76 (−1.4, −0.12)	0.92 (−1.07, 2.9)	0.034
Total sugar	−0.37 (−0.54, −0.2)	0.51 (−0.01, 1.03)	0.034
Energy (kJ/100 g)	−40.64 (−46.22, −35.06)	26.28 (5.8, 46.77)	0.034

^1^ based on Kruskal–Wallis test.

## Data Availability

All data supporting this research are provided within the manuscript and supplementary materials.

## Supplementary Materials

The following supporting information can be downloaded at: https://www.mdpi.com/article/10.3390/nu14122394/s1, File S1—Questions used to assess customer perceptions of intervention (yes or no responses); File S2—Plot of unit sales per week of food in control and intervention stores during 22 weeks of data collection (not during the intervention period), demonstrating similar overall patterns of unit sales in control and intervention stores.
