# Supplementary material for: Change in the Healthiness of Foods Sold in an Australian Supermarket Chain Following Implementation of a Shelf Tag Intervention Based on the Health Star Rating System
Source: Nutrients. 2022 Jun 9;14(12):2394. doi: 10.3390/nu14122394 (PMC9229209; doi:10.3390/nu14122394)

Supplementary File S1 – Questions used to assess customer perceptions of intervention (responses yes or no)

- Are you familiar with the new Health Star Rating System being introduced on the front of pack of many products?
- Did you notice shelf labels for all 4.5 and 5 star products in the store over the last month?
- In the last month, did you notice posters in the fruit/veg section telling you that all fresh fruit and vegetables are a 5 star healthy choice?
- Did the shelf labels and posters promoting healthier choices influence what you purchased from this store in the last month?

Supplementary File S2 – Plot of unit sales per week of food in control and intervention stores during 22 weeks of data collection (not during the intervention period), demonstrating similar overall patterns of unit sales in control and intervention stores.

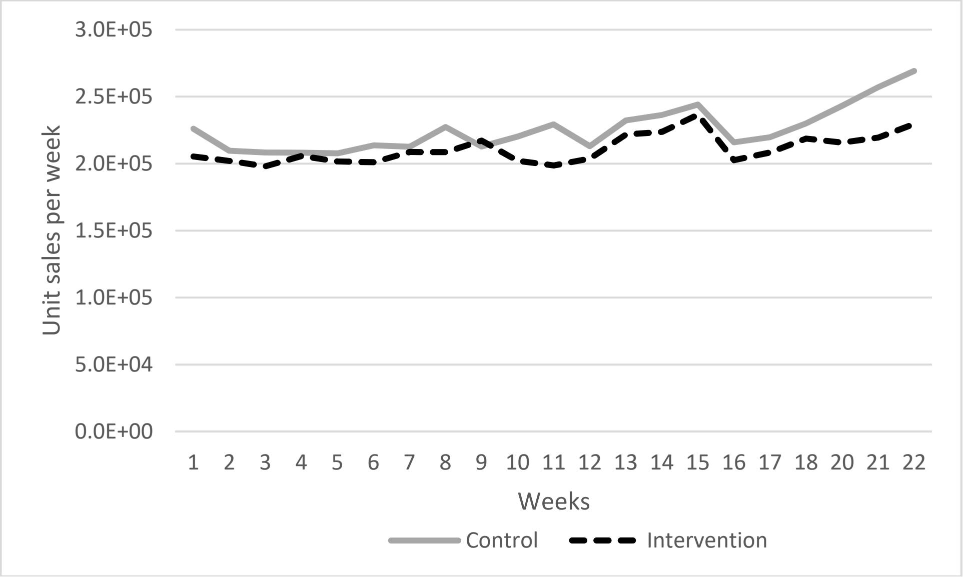

Supplement: Supplementary file 1 [file nutrients-14-02394-s001.zip › nutrients-1705304-supplementary.pdf]
